# Insect attachment on crystalline bioinspired wax surfaces formed by alkanes of varying chain lengths

**DOI:** 10.3762/bjnano.5.116

**Published:** 2014-07-14

**Authors:** Elena Gorb, Sandro Böhm, Nadine Jacky, Louis-Philippe Maier, Kirstin Dening, Sasha Pechook, Boaz Pokroy, Stanislav Gorb

**Affiliations:** 1 Department of Functional Morphology and Biomechanics, Zoological Institute, Kiel University, Am Botanischen Garten 9, D-24098 Kiel, Germany; 2 Department of Material Science and Engineering and the Russell Berrie Nanotechnology Institute, Technion – Israel Institute of Technology, 32000 Haifa, Israel

**Keywords:** *Coccinella septempunctata*, insect–plant interactions, plant waxes, pull-off force, traction force

## Abstract

The impeding effect of plant surfaces covered with three-dimensional wax on attachment and locomotion of insects has been shown previously in numerous experimental studies. The aim of this study was to examine the effect of different parameters of crystalline wax coverage on insect attachment. We performed traction experiments with the beetle *Coccinella septempunctata* and pull-off force measurements with artificial adhesive systems (tacky polydimethylsiloxane semi-spheres) on bioinspired wax surfaces formed by four alkanes of varying chain lengths (C_36_H_74_, C_40_H_82_, C_44_H_90_, and C_50_H_102_). All these highly hydrophobic coatings were composed of crystals having similar morphologies but differing in size and distribution/density, and exhibited different surface roughness. The crystal size (length and thickness) decreased with an increase of the chain length of the alkanes that formed these surfaces, whereas the density of the wax coverage, as well as the surface roughness, showed an opposite relationship. Traction tests demonstrated a significant, up to 30 fold, reduction of insect attachment forces on the wax surfaces when compared with the reference glass sample. Attachment of the beetles to the wax substrates probably relied solely on the performance of adhesive pads. We found no influence of the wax coatings on the subsequent attachment ability of beetles. The obtained data are explained by the reduction of the real contact between the setal tips of the insect adhesive pads and the wax surfaces due to the micro- and nanoscopic roughness introduced by wax crystals. Experiments with polydimethylsiloxane semi-spheres showed much higher forces on wax samples when compared to insect attachment forces measured on these surfaces. We explain these results by the differences in material properties between polydimethylsiloxane probes and tenent setae of *C. septempunctata* beetles. Among wax surfaces, force experiments showed stronger insect attachment and higher pull-off forces of polydimethylsiloxane probes on wax surfaces having a higher density of wax coverage, created by smaller crystals.

## Introduction

During their locomotion, insects use different structures for attachment, depending on the texture of the substrate. They usually apply their claws to interlock with surface irregularities on rough surfaces, when the diameter of the claw tip is smaller than the dimensions of typical surface asperities or cavities [1]. On smooth and microrough substrates, many insects use highly specialised adhesive pads, which may be located on different parts of the leg and are of two different types: smooth and setose (hairy) [2–3]. Due to the material flexibility of smooth pads and fine fibrillar surface microstructures (tenent setae) that cover hairy pads, both pad types can maximize the possible contact area with various substrate profiles (reviews [2,4–5]). Additionally, insect pads release a secretory fluid, which is most probably a micro-emulsion containing water-soluble and lipid-soluble fractions, onto a contact zone [2,6–8]. Due to such an elaborate system, insects are able to attach successfully and move efficiently on a variety of substrates (e.g., [9–11]) by using a broad range of physical interactions.

In nature, most insect species are associated with plants. During the long period of co-evolution between plants and insects, plants have developed surfaces that enable pollinators and symbiotic insects to attach to and walk on, as well as surface structures that reduce insect attachment [11]. The impeding effects of plant surfaces on insect attachment ability depend on the concrete plant–insect system and may serve as a defence mechanism against herbivores and nectar robbers or as a mechanism preventing the escape of insects from traps of carnivorous plants and kettle trap flowers. Plant surface features such as particular cell arrangements, shapes, and orientation, as well as the presence of some types of trichomes, acting mainly at the macroscopic level, hinder the interlocking of insect claws. Additionally, plant-produced wet films on the surface, microscopic cuticular folds and epicuticular (deposited onto the plant cuticle) wax crystals reduce the adhesion of insect attachment pads (reviews [11–12]). In the present study, we consider the effect of wax crystal dimension on insect attachment.

Three-dimensional projections, called wax crystals throughout the text, emerge from a two-dimensional film of cuticular lipids (waxes), representing the hydrophobic component of the plant cuticle [13]. Both projections and films exhibit a crystalline nature [14–15]. Wax crystals range in size from 0.5 to 100 μm and show various morphologies, such as platelets, rodlets, tubules, threads etc. [16–17], which originate from the self-assembly of specific molecules (e.g., [14,18–21]). The morphology of crystals is coherent with the chemical composition of the wax, representing a complex mixture of long-chain aliphatic and cyclic hydrocarbons, fatty acids, aldehydes, ß-diketones, primary and secondary alcohols, and is usually determined by the dominating chemical compound or compound class [16,22–23].

The effect of plant surfaces covered by wax crystals on insect attachment has been examined experimentally using different approaches in numerous previous studies (e.g., [10,24–44]). It has been demonstrated that such surfaces can greatly reduce the insect attachment ability when compared to wax free substrates. Not only the presence of wax crystals, but also their size and density affect insect attachment. The effect of the length and density of crystals has been observed in the case of *Cryptolaemus montrouzieri* beetles moving on the leaflets of the *Pisum sativum* plants covered with wild-type waxes and plant mutants with reduced wax coverage [40]. It has been found that the force reduction correlated with the increasing crystal length and the decreasing density of individual wax crystals. However, plant surfaces used also differed in the shape of the crystals in addition to their variability of dimension and density.

The aim of this study was to examine the effect of different parameters of crystalline wax coverage on insect attachment. To avoid the possible influence of chemical diversity and crystal shape, we decided, instead of using native plant wax surfaces, to use bioinspired wax surfaces covered by crystals having a similar morphology. Bioinspired surfaces were made of long-chain hydrocarbons, which can be dominating chemical constituents in plant waxes [22]. Four *n*-alkanes of varying chain lengths (C_36_H_74_, C_40_H_82_, C_44_H_90_, and C_50_H_102_) were used to form crystalline wax coatings having a different size and density of crystals. Different alkanes created different roughness on the surface [45–46].

Insect attachment ability was studied in traction experiments with adult seven-spotted ladybird beetles *Coccinella septempunctata* (Coleoptera, Coccinellidae) walking on five different substrates: four wax surfaces plus a hydrophilic smooth glass used as a reference sample. Two main questions were addressed. (i) Do insects perform differently on smooth glass and wax coated samples? (ii) How do different characteristics of crystalline wax surfaces influence the attachment? We also measured adhesion (pull-off) forces of artificial adhesive systems on these surfaces. Here, tacky and deformable polydimethylsiloxane (PDMS) semi-spheres, having elasticity moduli similar to those of insect adhesive pads were used as probes [47]. Results obtained in different types of force tests were compared and discussed from the perspective of insect attachment.

## Results

**Figure 1:**
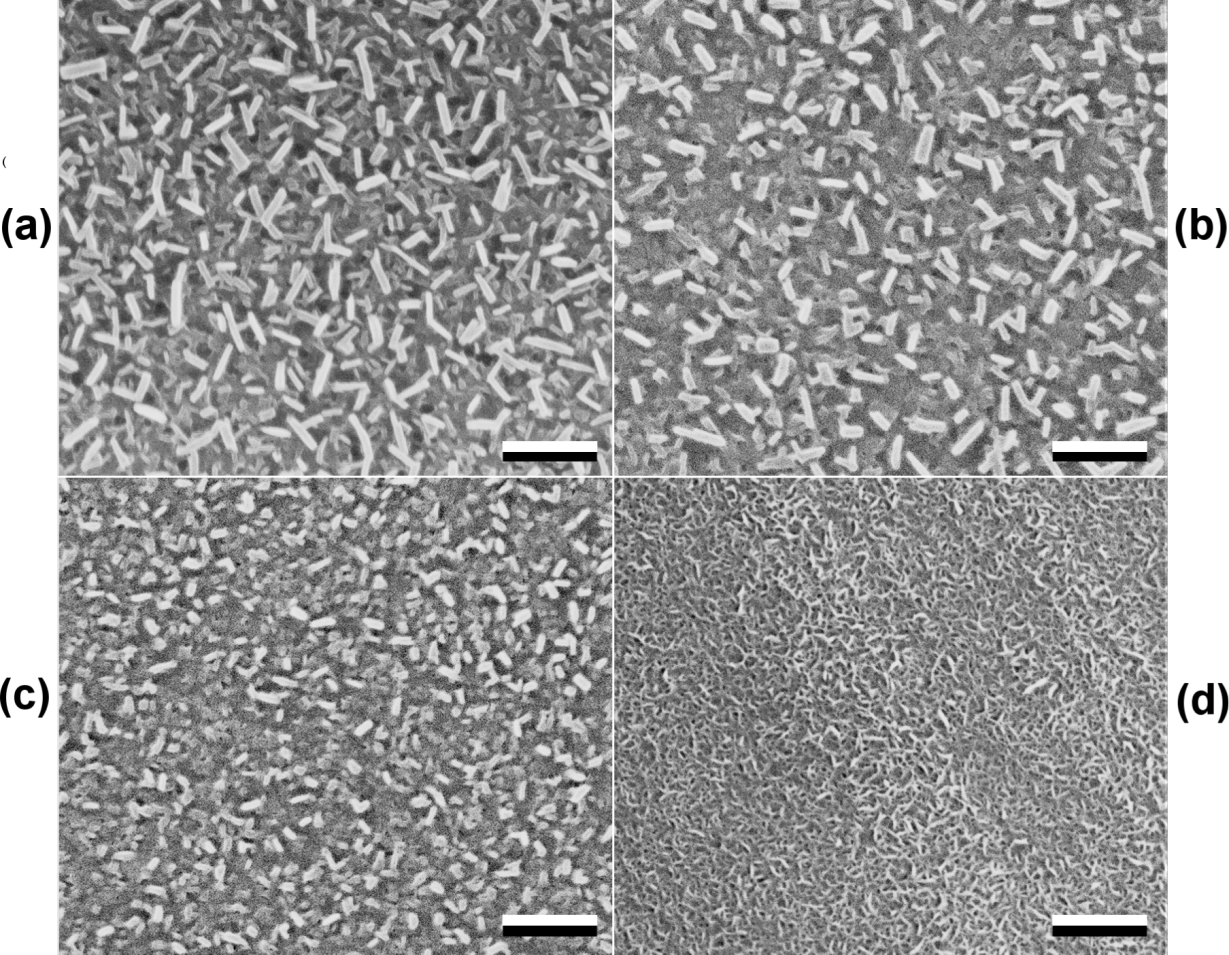
Scanning electron microscopy (SEM) micrographs of wax surfaces formed by alkanes of varying chain lengths: C_36_H_74_ (a), C_40_H_82_ (b), C_44_H_90_ (c), and C_50_H_102_ (d). Scale bars = 2 μm.

Bioinspired wax surfaces formed by alkanes of varying chain lengths (C_36_H_74_, C_40_H_82_, C_44_H_90_, and C_50_H_102_), referred to as C_36_, C_40_, C_44_, and C_50_, respectively, throughout the text, were regularly covered with submicroscopic plate-like wax crystals (Figure 1). The size of the wax crystals (Table 1) gradually decreased as the alkane chain length increased, showing a significant difference between surfaces (length: H_3,499_ = 336.512; thickness: H_3,499_ = 366.532; both *P* < 0.001, Kruskal–Wallis one way ANOVA on ranks). Also, pairwise comparisons of samples demonstrated significant differences in both morphometrical variables, with the exception of C_44_ vs C_50_ for the crystals length and C_36_ vs C_40_ for the crystals thickness (Table 2). On the contrary, the density of crystals on the surface (Table 1) showed a significant, up to five-fold, increase with an increased chain length (F_3,14_ = 359.201, *P* < 0.001, one way ANOVA). However, the density was similar in C_36_ and C_40_ (Table 2).

**Table 1:** Morphometrical variables of crystals, surface roughness, and wetting properties of wax samples.^a^

sample	CL, μm	CT, μm	CD, μm	*R*_a_, nm	r.m.s., nm	CA, °

C_36_	0.713 ± 0.213	0.170 ± 0.032	5.460 ± 0.617	973.66	1024.46	165.6 ± 1.5
	*n* = 125	*n* = 125	*n* = 4	*n* = 1	*n* = 1	*n* = 15

C_40_	0.483 ± 0.133	0.166 ± 0.032	4.956 ± 0.269	695.92	737.55	163.4 ± 1.1
	*n* = 125	*n* = 125	*n* = 4	*n* = 1	*n* = 1	*n* = 15

C_44_	0.301 ± 0.727	0.118 ± 0.022	8.386 ± 2.030	622.97	651.30	161.3 ± 1.9
	*n* = 125	*n* = 125	*n* = 3	*n* = 1	*n* = 1	*n* = 15

C_50_	0.279 ± 0.078	0.077 ± 0.013	27.786 ± 1.251	134.70	137.42	160.0 ± 2.2
	*n* = 125	*n* = 125	*n* = 4	*n* = 1	*n* = 1	*n* = 15

^a^CA, apparent contact angle of water; CD, density of crystals; CL, crystal length; CT, crystal thickness; *n*, number of individual measurements; *R*_a_, mean roughness; r.m.s., root mean square of roughness.

**Table 2:** Results of statistical analyses (Tukey test performed after Kruskal–Wallis one way ANOVA on ranks and one way ANOVA) of morphometrical variables of wax crystals between different wax samples.^a^

comparison	*q*	*P*	significantly different

***length***			
C_36_ vs C_40_	7.909	<0.05	yes
C_36_ vs C_44_	20.146	<0.05	yes
C_36_ vs C_50_	22.541	<0.05	yes
C_40_ vs C_44_	12.237	<0.05	yes
C_40_ vs C_50_	14.631	<0.05	yes
C_44_ vs C_50_	2.395	>0.05	no
***thickness***			
C_36_ vs C_40_	1.000	>0.05	no
C_36_ vs C_44_	12.954	<0.05	yes
C_36_ vs C_50_	23.364	<0.05	yes
C_40_ vs C_44_	11.955	<0.05	yes
C_40_ vs C_50_	22.264	<0.05	yes
C_44_ vs C_50_	10.409	<0.05	yes
***density***			
C_36_ vs C_40_	0.884	>0.05	no
C_36_ vs C_44_	4.760	<0.05	yes
C_36_ vs C_50_	39.101	<0.05	yes
C_40_ vs C_44_	5.578	<0.05	yes
C_40_ vs C_50_	49.987	<0.05	yes
C_44_ vs C_50_	31.443	<0.05	yes

^a^*P*, probability value; *q*, Tukey test statistics; yes, significantly different; no, no significant difference.

**Figure 2:**
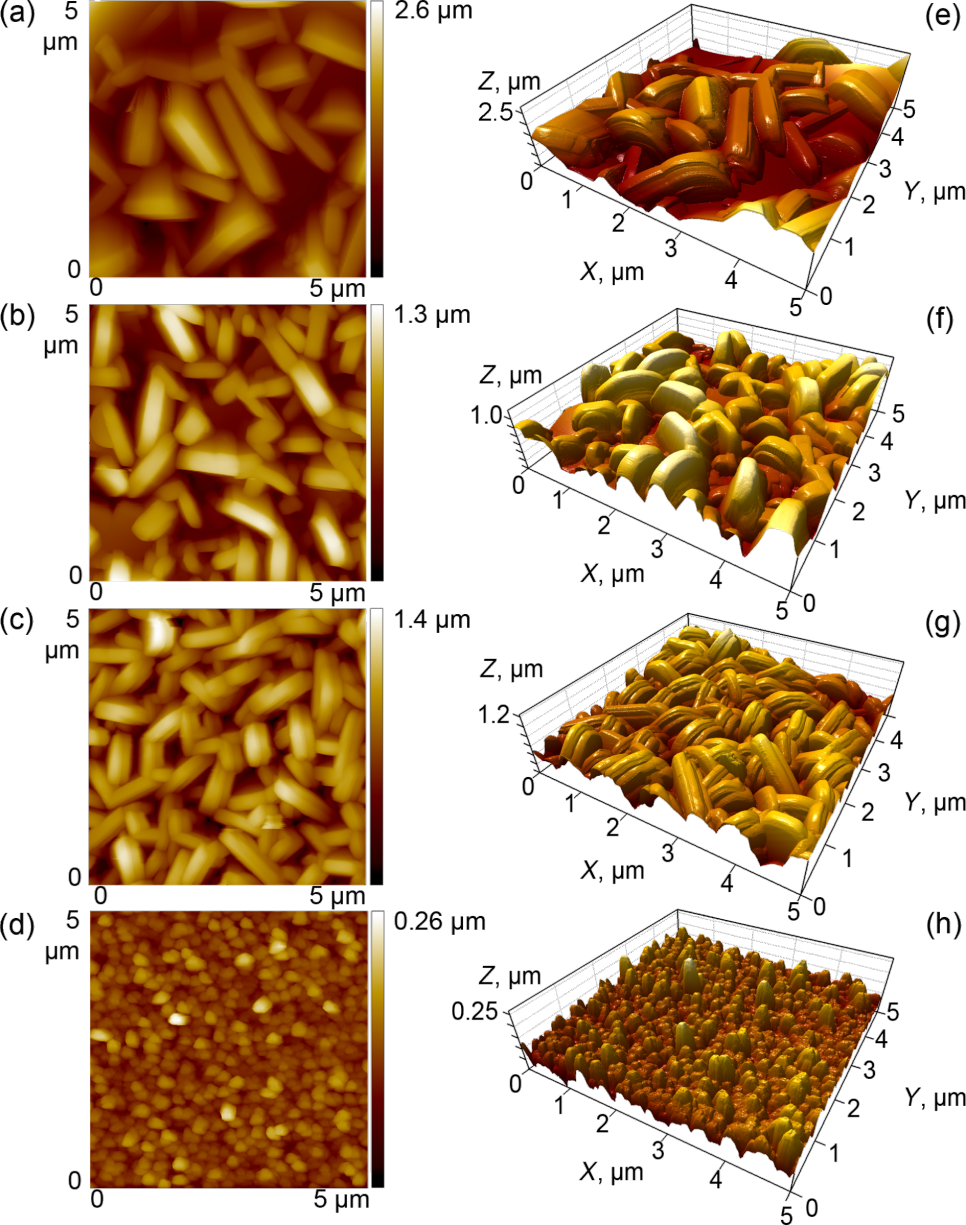
Atomic force microscopy (AFM) height images used for estimating the surface roughness parameters *R*_a_ and r.m.s. (a–d) and three-dimensional projections (e–h) of wax surfaces created by alkanes of varying chain lengths: C_36_H_74_ (a,e), C_40_H_82_ (b,f), C_44_H_90_ (c,g), and C_50_H_102_ (d,h).

Wax crystals created a microscopic and nanoscopic roughness of surfaces (Figure 2, Table 1). Values of the surface roughness parameters dropped by factors of 7–9, when C_36_ was compared with C_50._ Both middle-chain alkanes created surfaces with relatively similar mid-range roughness. All four wax samples showed superhydrophobic properties: apparent contact angles of water ranged from ca. 160 to 166° (Table 1).

**Figure 3:**
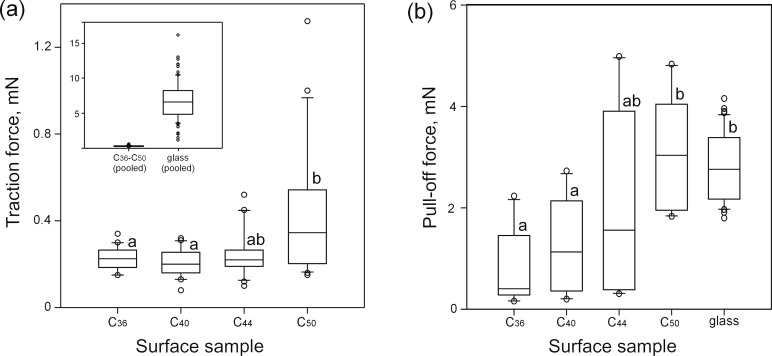
Traction forces of male beetles *Coccinella septempunctata* (a) and pull-off forces of PDMS semi-spheres (b) on different surfaces. According to the *t*-test and Mann–Whitney rank sum test (Table 3 and Table 4), means/medians with different letters differ significantly from each other.

The traction forces generated by beetles *Coccinella septempunctata* in the first and second tests on glass did not differ in either of the experimental sets with various wax surfaces (Table 3). Force values obtained on the glass sample were up to 30 times higher compared to those obtained on wax surfaces (Figure 3a, inset) and showed highly significant differences with the latter (Kruskal–Wallis one way ANOVA on ranks: H_4,159_ = 121.922, *P* < 0.001; Table 3). Traction forces measured on wax surfaces ranged from 0.224 ± 0.053 to 0.418 ± 0.301 mN. Comparisons between these samples revealed a trend toward an increase of force values in the order of surfaces C_36_–C_40_–C_44_–C_50_ (Figure 3a): Forces on the C_50_ surface were significantly higher compared to those on C_36_ and C_40_ surfaces, whereas forces in other surface pairs were similar (Table 3).

**Table 3:** Results of statistical analyses (*t*-test, Mann–Whitney rank sum test, and Dunn’s method performed after Kruskal–Wallis one way ANOVA on ranks) of traction force values obtained on different samples.^a^

comparison	d.f.	test statistics	*P*	significantly different

***glass: 1 vs 2***				
C_36_	38	*t* = 0.048	0.962	no
C_40_	—	*T* = 428	0.636	no
C_44_	38	*t* = 0.580	0.565	no
C_50_	—	*T* = 4040	0.862	no
***glass vs wax***				
C_36_	—	*Q* = 7.203	<0.05	yes
C_40_	—	*Q* = 7.743	<0.05	yes
C_44_	—	*Q* = 6.938	<0.05	yes
C_50_	—	*Q* = 5.734	<0.05	yes
***between wax***				
C_36_ vs C_40_	38	*t* = 1.105	0.312	no
C_36_ vs C_44_	—	*T* = 396	0.715	no
C_36_ vs C_50_	—	*T* = 319	0.015	yes
C_40_ vs C_44_	—	*T* = 362	0.198	no
C_40_ vs C_50_	—	*T* = 299	0.003	yes
C_44_ vs C_50_	—	*T* = 340	0.062	no

^a^d.f., degrees of freedom; *P*, probability value; *Q*, Dunn’s method statistics; *t*, t-test statistics; *T*, Mann–Whitney rank sum test statistics; yes, significantly different; no, no significant difference.

Pull-off forces measured using PDMS semi-spherical probes were either significantly lower in cases of shorter-chain alkanes or similar in cases of longer-chain alkanes, when compared with forces obtained on the glass sample (Figure 3b, Table 4). Among the wax surfaces, we observed a trend of rising pull-off forces in the following order of samples C_36_–C_40_–C_44_–C_50_ (Figure 3b): the lowest force was measured on C_36_ (0.800 ± 0.712 mN) and the highest one on C_50_ (3.092 ± 1.094 mN). However, significant differences in force values were found only in C_50_ vs C_36_ and C_50_ vs C_40_ (Table 4). In other pairs of surfaces, differences were non-significant (Table 4).

**Table 4:** Results of statistical analyses (*t*-test and Mann–Whitney rank sum test) of pull-off force values obtained on different samples.

comparison	d.f.	test statistics	*P*	significantly different

***glass vs wax***				
C_36_	48	*t* = 8.733	<0.001	yes
C_40_	48	*t* = 6.317	<0.001	yes
C_44_	—	*T* = 197	0.163	no
C_50_	—	*T* = 268	0.762	no
***between wax***				
C_36_ vs C_40_	18	*t* = −1.252	0.226	no
C_36_ vs C_44_	—	*T* = 81	0.076	no
C_36_ vs C_50_	—	*T* = 58	<0.001	yes
C_40_ vs C_44_	—	*T* = 92	0.345	no
C_40_ vs C_50_	18	*t* = −4.082	<0.001	yes
C_44_ vs C_50_	18	*t* = −1.467	0.160	no

d.f., degrees of freedom; *P*, probability value; *t*, t-test statistics; *T*, Mann–Whitney rank sum test statistics; yes, significantly different; no, no significant difference.

**Figure 4:**
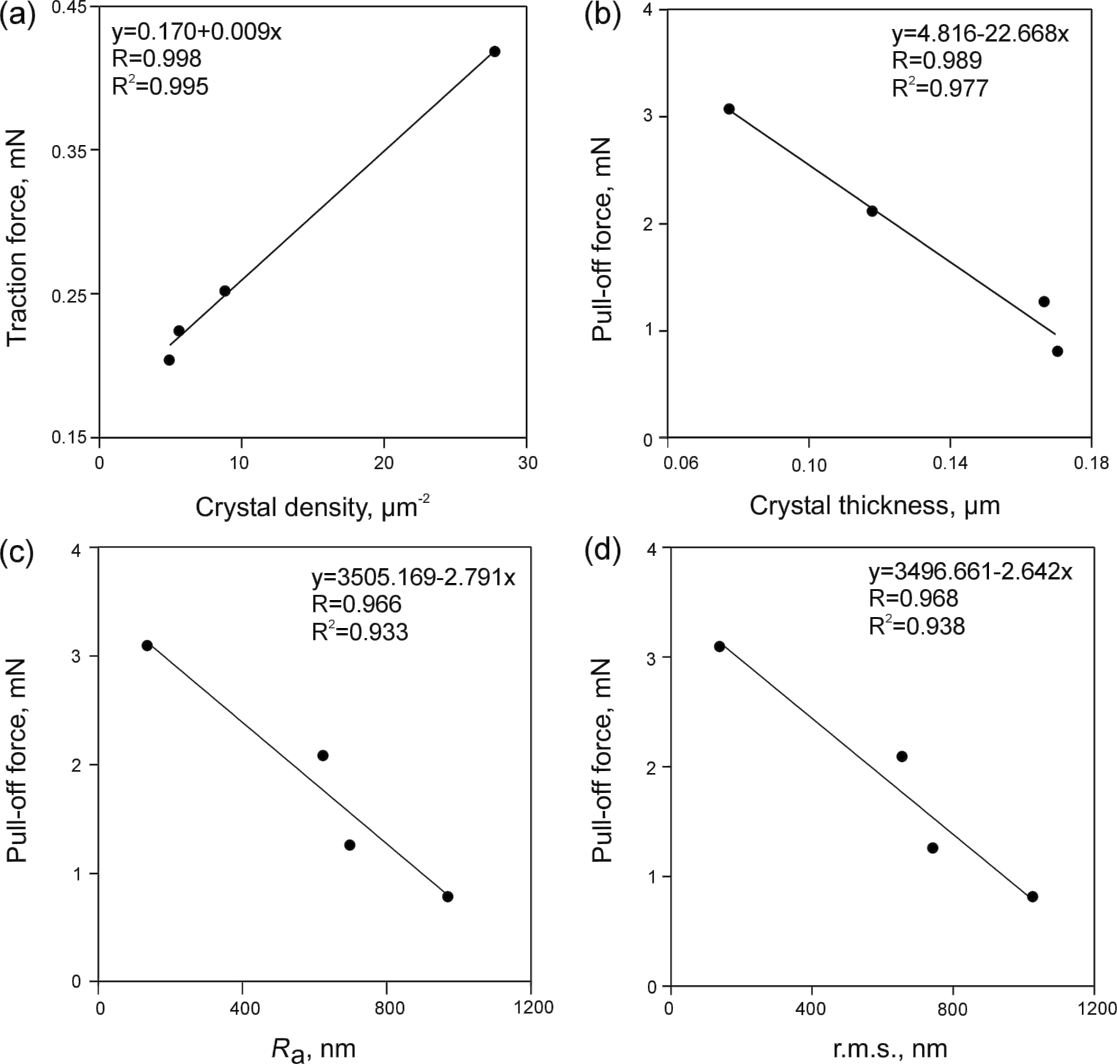
Traction forces of male beetles *Coccinella septempunctata* vs crystal densities (a) and pull-off forces of PDMS semi-spheres vs crystals thickness (b), mean roughness *R*_a_ (c), and root mean square of roughness r.m.s. (d). Lines indicate linear regressions.

Correlations between forces and different wax surface parameters (crystal length, crystal thickness, density of crystals, mean roughness_,_ and root mean square of roughness) were examined. We found a significant positive correlation between the traction force and crystal density (*P* = 0.002, linear regression; Figure 4a). The pull-off force showed significant negative correlations with the crystal thickness (*P* = 0.011, linear regression; Figure 4b), mean roughness *R*_a_ (*P* = 0.034, linear regression; Figure 4c), and root mean square of roughness r.m.s. (*P* = 0.032, linear regression; Figure 4d). Other correlations between forces and wax surface parameters were non-significant (*P* > 0.05, linear regression).

## Discussion

Using *n*-alkanes of varying chain lengths (C_36_H_74_, C_40_H_82_, C_44_H_90_, and C_50_H_102_), we obtained surfaces with crystalline wax coatings composed of crystals having similar plate-like shapes. Wax crystals were either of the same dimensions as epicuticular crystals in plants [16–17] or even smaller, because we used alkanes with the same or longer chain lengths, when comparing to plant wax compounds (20 to 40 carbons [22–23]). Microscopy studies of our bioinspired wax surfaces showed differences in both crystal size and density among the samples: The length and thickness of the crystals decreased with an increase of the chain length of the alkanes that formed these surfaces, whereas the density of the wax coverage showed an opposite relationship. These differences in the wax coverage micromorphology caused distinctions in the surface roughness. Samples bearing looser coverage were composed of larger (longer and thicker) crystals and were rougher than samples with smaller (shorter and thinner) crystals covering the surface more densely. Since the surface roughness dropped in the order of surfaces C_36_–C_40_–C_44_–C_50_, it demonstrated a similar dependence on the chain length as did the morphometrical variables of crystals. Interestingly, the values of surface roughness parameters measured using AFM on the crystalline epicuticular wax in the pitcher of the *Nepenthes alata* plant (*R*_a_ = 0.254 ± 0.035 μm, r.m.s. = 0.317 ± 0.045 μm [48]) were in the range of those measured in our bioinspired samples, to be exact between those of C_44_ and C_50_ samples. The fine microroughness of bioinspired surfaces covered by hydrophobic wax material led to very high values of the apparent contact angle of water, which were very close to those measured on plant surfaces bearing three-dimensional waxes (e.g., [49–51]).

**Figure 5:**
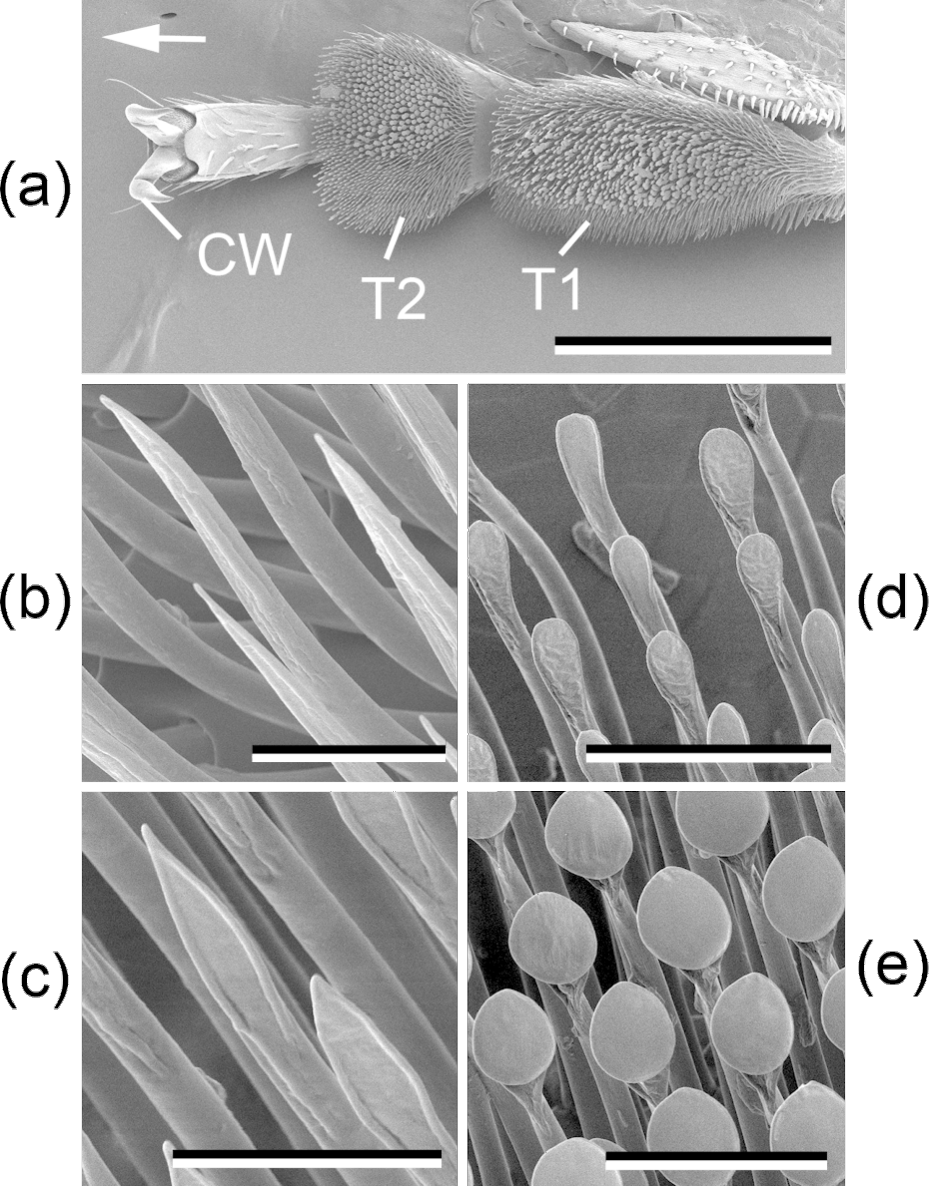
SEM micrographs of tarsal attachment devices in the male beetle *Coccinella septempunctata*: tarsus of the foreleg (a) and different types of tenent setae (b–e). Arrow in (a) shows the distal direction. CW, claw; T1, first proximal tarsomere; T2, second proximal tarsomere. Scale bars = 500 μm (a), 20 μm (d,e), 10 μm (b,c). Adapted from [52].

The tarsal attachment system of the *C. septempunctata* beetle used in this study has been previously described in detail by Gorb et al. [52]. The tarsus bears two ventrally curved claws with a claw tip diameter of about 4 μm [42] and hairy adhesive pads situated on the ventral side of the two first proximal tarsomeres (Figure 5a). Pads are covered with numerous tiny setae having various tip shapes, from sharp-pointed to spatula-like, ranging in width from ca. 1.8 to 3.5 μm (Figure 5b–d). Male beetles additionally have large areas (up to a half of the total pad area) covered by setae with discoid terminal elements having an average diameter of 8 μm (Figure 5a,e). These setae are adapted for holding strongly on to females for a long time during mating [53–54].

As the studied substrates were lacking surface structures suitable for claw interlocking (larger than 4 μm according to [1]), we assume that insect attachment relied solely on the performance of adhesive pads. Traction force tests demonstrated a great reduction in beetle attachment on microstructured crystalline wax surfaces compared to smooth glass (*R*_a_ = 0.007 ± 0.001 μm, r.m.s. = 0.009 ± 0.001 μm [47]). These results are in line with previous experimental data obtained for different insect species on three-dimensional plant waxes vs smooth surfaces (see Introduction). The good performance of the beetles during the second run on glass, which followed the test on each bioinspired wax surface, indicated no influence of the wax coatings on the subsequent attachment ability of insects. This may also suggest that there was no contamination of insect adhesive organs by wax crystals in our experiments, although a contaminating effect caused by plant crystalline waxes has been repeatedly reported previously (e.g., [25,38,55–57]). Therefore, we did not consider contamination as a mechanism for the reduction of beetle attachment on bioinspired wax surfaces studied (see hypotheses proposed for plant waxes [10]). The obtained data may be primarily explained by the inability of relatively large setal tips to make full contact with these surfaces: The real contact area was highly reduced presumably due to the micro- and nanoscopic roughness introduced by wax crystals (roughness hypothesis according to [10]). The impact of the surface roughness on insect adhesion has been shown in a number of experimental studies demonstrating a greatly reduced adhesion on the surface roughness of specific microscopic dimensions in comparison to smooth substrate [2,58–60]. Also, an adsorption of the secretion from insect adhesive pads by the bioinspired wax coverage and/or wax dissolving in the pad secretion leading to hydroplaning cannot be completely excluded (adsorption hypothesis and wax dissolving hypothesis [10]).

Using PDMS semi-spheres as artificial adhesive systems, we obtained much higher forces on wax samples compared to insect attachment forces measured on these surfaces. These force values were either close or equal to those measured on glass. We explain these results by differences in the material properties between PDMS and tenent setae of *C. septempunctata* beetles. The PDMS probes exhibited elastic properties (effective E-modulus of ca. 70 kPa), which were in the range of those in smooth adhesive pads of insects (27 kPa in *Tettigonia viridissima* [61] and 12–625 kPa in *Carausius morosus* [62]). Due to high deformability of the material, PDMS probes were able to make good contact with the substrates. Similar force values obtained on glass and on both C_50_ and C_44_ suggest a similar real contact area between the probes and these three surfaces. The material in the hairy adhesive pads of *C. septempunctata* is much stiffer: The lowest E-modulus recently measured at the setal tip is 1 MPa [63]. Therefore, the pressing in of less deformable pad material into cavities between the wax crystals probably did not occur, resulting in a smaller real contact area and, consequently, lower traction forces. Also, the lack of pressing in will not lead to the mechanical interlocking at the interface and will not contribute to increase of traction forces.

Among the bioinspired wax surfaces, the highest attachment force was measured on C_50_. It significantly correlated with the densest coverage, created by the smallest crystals. These results are not in line with our previous findings showing the opposite effects of the crystal size and density on insect attachment [40]. The discrepancy may be due to differences in the micromorphology of the crystals between the surfaces used in these studies. Here, all wax surfaces were uniformly covered with platelet-like crystals, whereas previous data were obtained on real plant surfaces bearing crystals of diverse shapes (platelets, combination of scales with ribbons, and combination of filaments with ribbons and rodlets).

Pull-off force measurements performed on bioinspired wax surfaces with artificial adhesive systems also showed the highest force on the C_50_ surface. Force values grew with a decrease in both crystal sizes (thickness) and surface roughness. The latter result is in accordance with data of previous studies on rubber surfaces, where the highest forces were recorded on substrates with the finest microroughness [47,64–65]. In these cases, as well as in C_50_, the small dimensions of the surface structures together with their dense distribution on the surface resulted in a rather smooth surface topography. Such substrate profiles can be replicated by very deformable material down to a micro- or even nanometer scale due to high flexibility of one of the contact partners (substrate or probe).

## Conclusion

By using *n*-alkanes of varying chain lengths, we obtained highly hydrophobic surfaces with wax coatings composed of crystals having similar shapes, but differing in size and distribution/density. Insect attachment on these substrates probably relied solely on the performance of adhesive pads. Force experiments showed stronger insect attachment ability and higher pull-off forces of PDMS probes on the wax surface with a higher density of wax coverage, created by smaller (thinner) crystals. At this (sub)microscopic range of roughness, adhesion grew with an increase of the substrate smoothness.

## Experimental

Wax surfaces were prepared through self-assembly of *n*-alkane hexatriacontane C_36_H_74_ (98%), tetracontane C_40_H_82_ (≥95%), tetratetracontane C_44_H_90_ (99%), and pentacontane C_50_H_102_ (≥97%) (Sigma-Aldrich, France). The waxes were deposited by thermal evaporation using a Bio-Rad Polaron Division Coating System, in a vacuum chamber at 10^−4^ mbar on a holder placed 10–12 cm above a crucible loaded with 40–50 mg of wax. The wax was evaporated at a crucible temperature of about 200 °C by applying pulses of an electrical current. After evaporation, the specimens were kept at room temperature (25 °C).

Surface imaging was performed by using SEM (FEI E-SEM Quanta 200, FEI Company, Hillsboro, OR, USA). Morphometrical variables of wax crystals (length CL and thickness CW) and density of crystals (CD) covering the surface of bioinspired wax samples were measured from digital images using the image analysis software SigmaScan Pro 5.0.0 (SPSS Inc., Chicago, IL, USA).

The topography of wax surfaces was examined using the AFM (Typ NanoWizard, JPK Instruments AG, Berlin, Germany). On each sample, areas of 5 μm × 5 μm were scanned in intermittent contact mode with a supersharp silicon non-contact cantilever (SSS-NCH, Nanoworld, Neuchâtel, Switzerland). The scanned area was comparable with the size of setal tips in adhesive pads of the beetle *C. septempunctata* [52]. The images obtained were processed with the scanning probe image processing software (SPIP, Version 5.1.2, Image Metrology A/S, Hørsholm, Danemark) for evaluation of roughness parameters, such as *R*_a_ (mean roughness) and r.m.s. (root mean square of roughness). Prior to roughness analyses, the images were plane corrected. The scanning tip geometry was characterized by scanning on a mica disc covered with colloidal gold particles (5.5, 9.3, and 14.4 nm in diameter) (Pelco AFM Gold standard Kit Product No. 16205, TED Pella Inc., Redding, CA, USA). Using the SPIP software, the radius of 13 nm was determined via blind tip reconstruction.

Measurements of contact angles of double-distilled water on wax surfaces were performed by using a high-speed optical contact angle measuring device OCAH 200 (DataPhysics Instruments GmbH, Filderstadt, Germany) according to the needle-in sessile drop method. For a detailed description of the method, see [50]. We applied 1 μL drops and ellipse fitting for evaluation of apparent contact angles. On each surface, the contact angles of 15 drops were measured. Altogether, 60 measurements were executed.

To measure the attachment forces of *Coccinella septempunctata* beetles on different substrates, traction experiments with tethered walking male insects were carried out by using a load cell force transducer (10 g capacity, Biopac Systems Ltd, Santa Barbara, CA, USA) as described in [52]. Insects were collected from plants along roadsides near Kronshagen (Germany). The experimental design includes three successive force tests with the same beetle: first on hydrophilic smooth glass (glass 1), then on one of the wax surfaces, and once more on glass (glass 2). Each insect individual was tested on all wax surfaces. We used 20 male beetles and carried out 240 traction tests in total.

Pull-off forces were measured in indentation experiments with the micro-tribometer Basalt 01 (Tetra GmbH, Ilmenau, Germany), which has been described in detail in [66]. We applied a recently developed technique for testing surfaces with very low adhesive capability by using elastic (effective E-modulus of ca. 70 kPa) PDMS semi-spheres as probes [47]. With each probe, pull-off force measurements were conducted at an applied normal force of ca. 1 mN on two samples: (1) hydrophilic smooth glass and (2) one of the wax surfaces. The order of samples was not randomized. We used 40 probes (20 for each wax surface) and performed 80 pull-off force measurements in all.

All experiments were carried out at room temperature (20–25 °C) and a relative ambient humidity of 30–42%. Values are presented in the text as “mean ± standard deviation”. Data obtained were statistically analysed with the SigmaStat 3.5 software (SPSS Inc., Chicago, IL, USA). One way ANOVA and Kruskal–Wallis one way ANOVA on ranks were used to evaluate differences in the morphometrical variables of the crystals between wax surfaces. We applied Kruskal–Wallis one way ANOVA on ranks for comparison of traction force values on glass and wax surfaces. To compare traction forces and pull-off forces obtained on pairs of different substrates, data were analysed with *t*-test and Mann–Whitney rank sum test. Correlations between forces and different wax surface parameters (crystal length CL, crystal thickness CT, density of crystals CD, mean roughness *R*_a,_ and root mean square of roughness r.m.s.) were examined by using linear regression.
